# Radiation recall phenomenon after administration of the mRNA-1273 SARS-CoV-2 vaccine

**DOI:** 10.1007/s13691-021-00528-5

**Published:** 2022-01-09

**Authors:** Yojiro Ishikawa, Rei Umezawa, Takaya Yamamoto, Noriyoshi Takahashi, Kazuya Takeda, Yu Suzuki, Keiichi Jingu

**Affiliations:** Department of Radiation Oncology, Tohoku University Graduate School of Medicine, 1-1 Seiryo-chou, Aoba-ku, Sendai, 980-8574 Japan

**Keywords:** SARS-CoV-2 vaccine, Radiation recall phenomenon, Breast cancer

## Abstract

Several types of SARS-CoV-2 vaccine have been developed. However, the relationship between SARS-CoV-2 vaccine and radiation therapy (RT) is unclear. Recently, there have been some reports of radiation recall phenomenon (RRP) caused by a SARS-CoV-2 vaccine. We report a case of RRP after administration of the mRNA-1273 SARS-CoV-2 vaccine. A 51-year-old female was diagnosed with breast cancer (cT4N1M0, cStage IIIB) and underwent breast total mastectomy with axillary lymph node dissection after neoadjuvant chemotherapy. After mastectomy, the patient received RT with 50 Gy in 25 fractions. An acute side effect of grade 2 dermatitis according to the National Cancer Institute Common Terminology Criteria for Adverse Events version 4.0. occurred after RT. The patient had not started any new systemic medication after RT; however, the patient received the mRNA-1273 SARS-CoV-2 vaccine (Moderna) 1 month after the end of the initial RT. Seven days after vaccination, the patient had a skin reaction with burning sensation and redness. This skin reaction was induced in an area corresponding to the irradiation field of the chest wall. There was no skin reaction in areas other than that described. The reaction was cured within 1 week with topical hydrocortisone. This report is an interesting case report with a RPP after administration of the mRNA-1273 SARS-CoV-2 vaccine.

## Introduction

SARS-CoV-2 vaccines have been developed by various companies including Pfizer-BioNTech, Moderna and AstraZeneca. When inoculating, it is necessary to have sufficient risk communication with the recipient and to inoculate intramuscularly with an appropriate method. However, the long-term efficacy and safety of all vaccines are not clear, and their relationship with radiation therapy (RT) is unclear.

Radiation recall phenomenon (RRP) is defined as a phenomenon in which an inflammatory response is induced in an area corresponding to the irradiation field by administering a specific drug to a patient who has a history of radiotherapy (RT) [1]. RRP does not develop slowly as in radiation dermatitis but develops rapidly.

There are some reports of the RRP caused by the SARS-CoV-2 vaccine [2–4]. To the best of our knowledge, this is the first report of RRP after administration of a mRNA-1273 SARS-CoV-2 vaccine (manufactured by Moderna).

## Case report

**Fig. 1:**
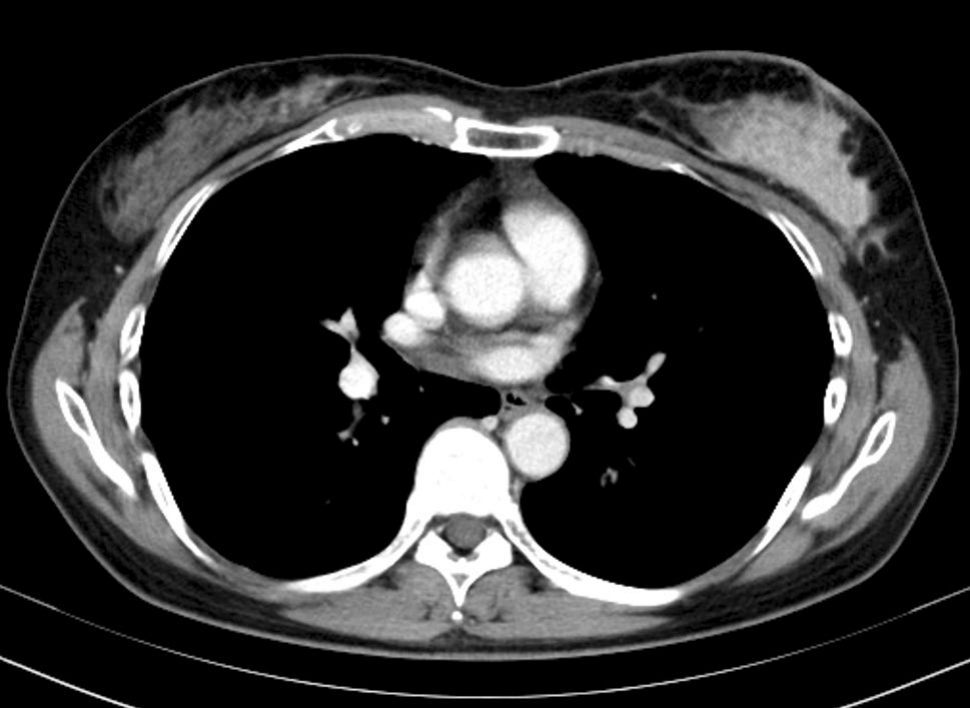
CT scan revealed a mass lesion measuring 62 mm in size in the left breast

A 51-year-old female presented with lumpiness and nipple depression in her left breast. The patient had no medical history or family history of breast or ovary cancer; however, the patient had some allergies to fruits. There was no history of drinking or smoking. At the previous hospital, a CT scan revealed a mass lesion measuring 62 mm in size in the left breast and swelling two axillary lymph nodes (Fig. 1). There was no distant metastasis. A core needle biopsy revealed invasive ductal carcinoma (IDC). Based on these examinations, the patient was diagnosed with breast cancer (cT4N1M0, cStage IIIB) and underwent breast total mastectomy with axillary lymph node dissection after neoadjuvant chemotherapy. Microscopic findings after resection showed that the tumor was IDC, estrogen receptor-negative, progesterone receptor-negative, human epidermal growth factor receptor 2 (HER2)-negative and the value of Ki-67 index was 52% (ypT3N2a, cM0, ypStage IIIA).

**Fig. 2:**
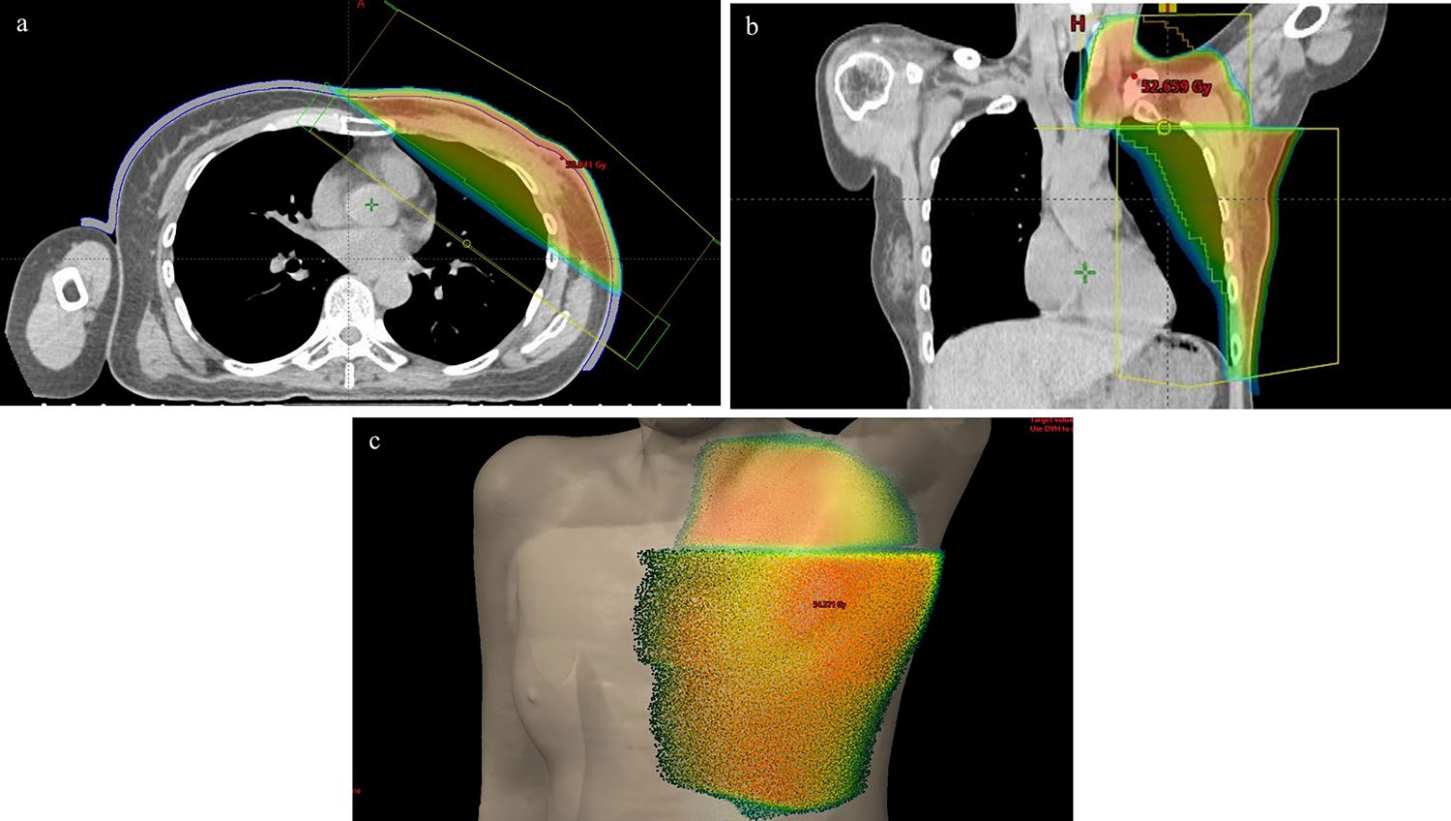
Dose distribution of radiation therapy in an axial image (**a**) and coronal image (**b**). The left chest wall and subclavian lymph node area were treated with 50 Gy in the skin surface (**c**)

The patient was administered capecitabine (2500 mg/m^2^/day) because the patient had a residual tumor of HER2-negative breast cancer after neoadjuvant chemotherapy. After mastectomy, the patient received RT with 50 Gy in 25 fractions of initial irradiation for the left chest wall and lymph node area. The chest wall tumor was treated with 6 MV photon fields with 0.5 cm bolus (Fig. 2).

**Fig. 3:**
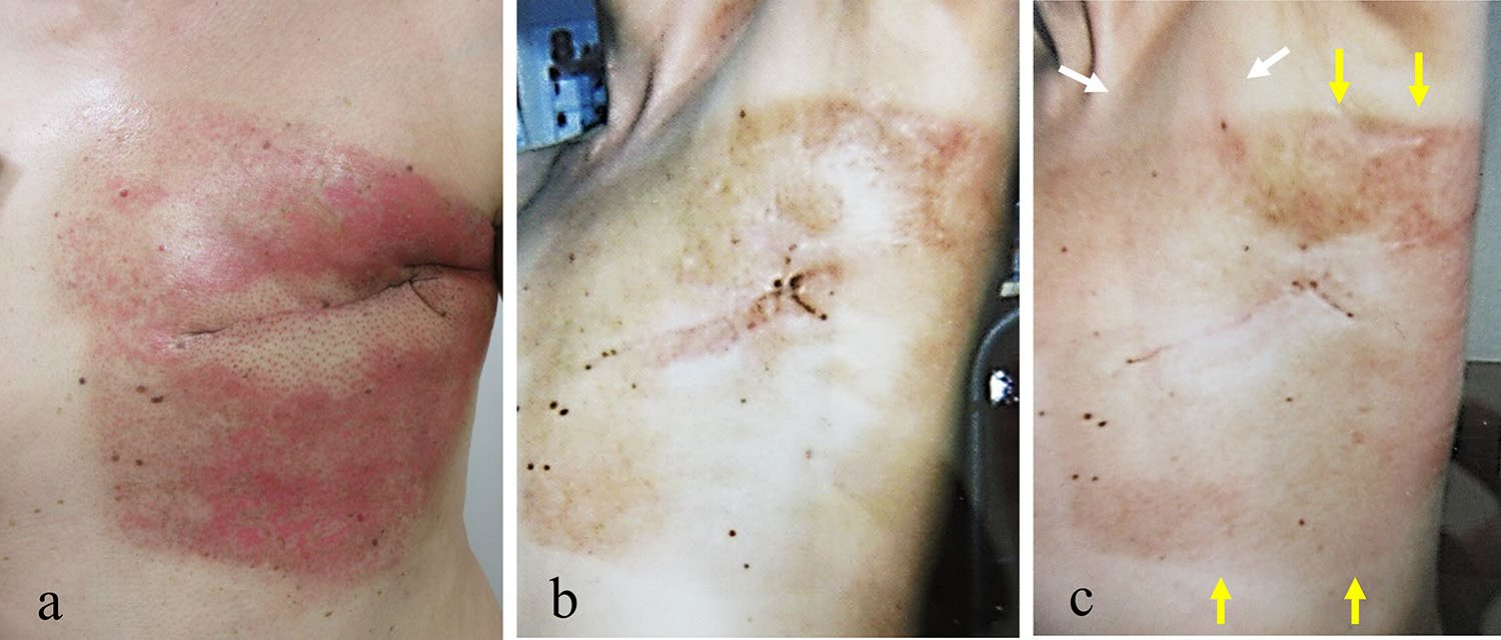
Acute side effect of grade 2 dermatitis in the left chest wall according to the National Cancer Institute Common Terminology Criteria for Adverse Events version 4.0. occurred after radiotherapy (**a**). Macroscopical findings showed that some pigmentation remained 1 month after radiation therapy (before vaccination) (**b**). Seven days after vaccination, mild redness of the demarcated skin consistent with the irradiation field of the left chest wall occurred after radiotherapy as judgment by two radiation oncologists (yellow arrow). There was also slight redness in the left subclavian lymph node area (white arrow) (**c**)

An acute side effect of grade 2 dermatitis according to the National Cancer Institute Common Terminology Criteria for Adverse Events version 4.0. occurred after RT (Fig. 3a). The dermatitis was cured within 1 month with topical hydrocortisone (Fig. 3b).

**Fig. 4:**
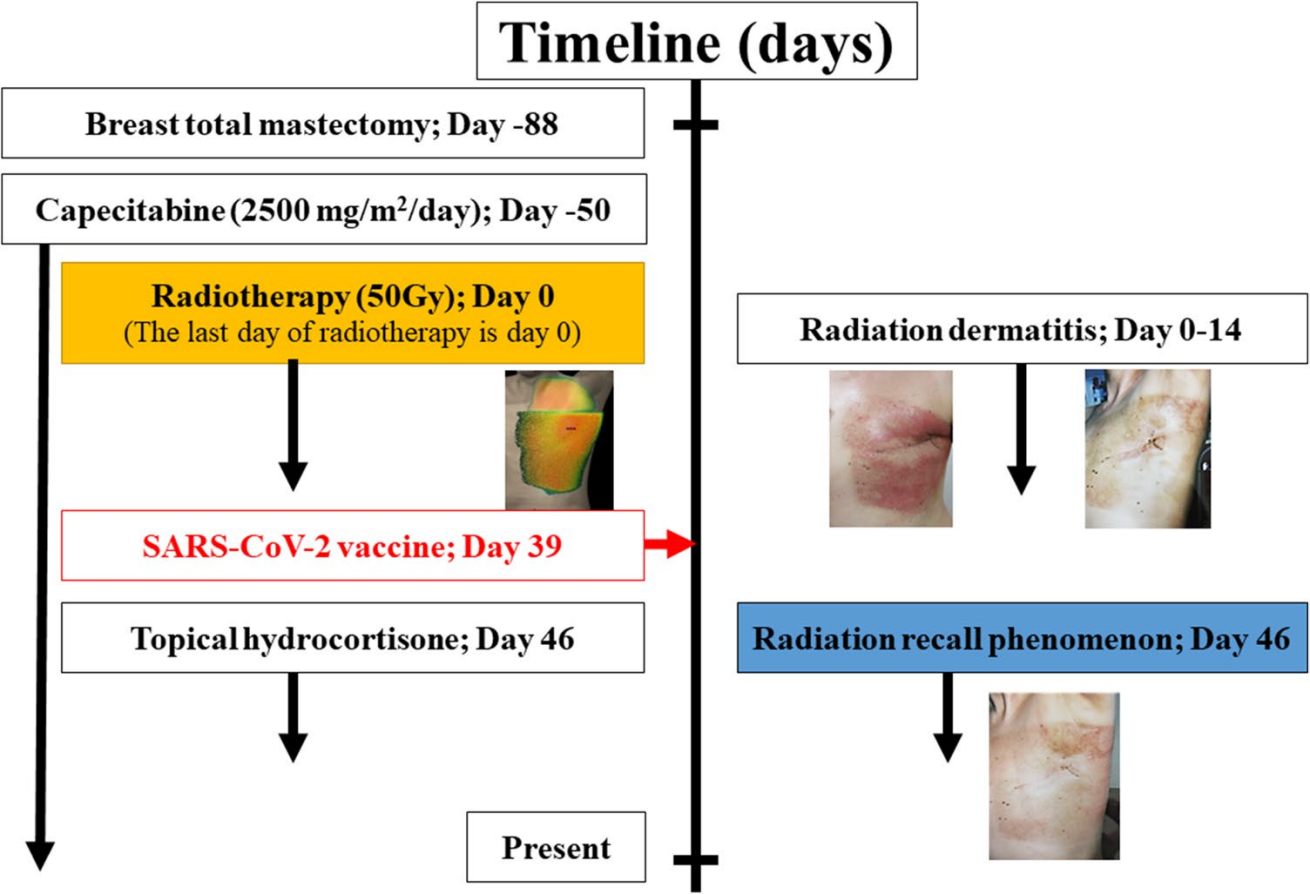
Timeline of radiation recall phenomenon

The patient had not started any new systemic medication after RT; however, the patient received the mRNA-1273 SARS-CoV-2 vaccine (manufactured by Moderna) 1 month after the end of the initial RT. Fifteen minutes after vaccination, the patient complained of itching in the throat and rash on both lower extremities. These symptoms disappeared with conservative treatment. Seven days after vaccination, the patient had a fever followed by a skin reaction with burning sensation, redness and slight skin exfoliation in the light chest and infra and supra clavicular area (Fig. 3c). There was no skin reaction in areas other than that described. The reaction was cured within 1 week with topical hydrocortisone. Capecitabine was continued to be administered after the dermatitis occurred. These symptoms disappeared with conservative treatment. Seven days after vaccination, the patient had a fever followed by a skin reaction with burning sensation, redness and slight skin exfoliation in the light chest and infra and supra clavicular area, judgment by two radiation oncologists. There was no severe myelosuppression during capecitabine administration. The patient refused to receive a second vaccination due to concerns about side effects of the vaccine. After the dermatitis disappeared, we consulted a dermatologist at our hospital for dermatitis of the left chest wall. The dermatologist’s examination ruled out dermatitis caused by common diet or medication. This sequence of RT and vaccinations is detailed in the timeline (Fig. 4).

## Discussion

Since D'Angio reported RRP caused by D-actino maycin in 1959, various inducers have been reported [1]. Most of the inducers of RRP are chemotherapy agents; however, there are reports of RRP caused by anti-tuberculosis drugs and ultraviolet rays [5, 6]. Reports of RRP caused by chemotherapy agents such as taxanes and anthracyclines account for 20–30% of the cases of RRP [7]. There is also a theory that the dose of radiation therapy, the dose of the drug, and the timing of drug administration are involved in the induction [8]. If the drug is used continuously after the appearance of RRP, there is a high possibility that RRP will recur, and steroids and histamines antagonist may be pre-administered [9].

According to the literature, there have been five cases of RPP associated with a SARS-CoV-2 vaccine (Table 1), but RPP caused by the SARS-CoV-2 vaccine itself remains a poorly understood phenomenon. Soyfer et al. reported two cases of RRP after administration of the Pfizer-BioNTech mRNA vaccine for a SARS-CoV-2 vaccine [4]. In addition, two cases with dermatitis of RRP occurred after administration of the Sinovac vaccine of inactivated virus vaccine and AstraZeneca of vector vaccine [3, 10]. To the best of our knowledge, this is the first report of dermatitis of RRP after administration of the mRNA-1273 SARS-CoV-2 Vaccine (manufactured by Moderna). On the other hand, a case of pneumonitis of RRP after administration of the Moderna mRNA vaccine was reported by Steber et al. [2].

**Table 1:** Radiation recall phenomenon after administration of SARS-CoV-2 vaccine

Report	Age	sex	Type of vaccine	Vaccinations	primary tumor	Tumor site	Radiation dose (to tumor) (Gy)	Time interval (end of radiotherapy to 1st vaccination)	Time to onset of skin reaction (final vaccination to skin reaction)	Severity of skin reaction
Soyfer V et al.	68	male	Pfizer-BioNTech (mRNA vaccine)	Twice	Unclassified spindle cell sarcoma	Back	50	175 days	5 days	Mild
Soyfer V et al.	64	Male	Pfizer-BioNTech (mRNA vaccine)	Twice	Solitary fibrous tumor	Chest wall	39	11 days	6 days	Mild
Afacan E et al.	60	Female	Sinopharm (inactivated virus vaccine)	Twice	Melanoma	Leg	30	39 months	5 days	Mild
Stewart R et al.	57	Female	AstraZeneca (vector vaccine)	Once	Acinic cell carcinoma	Parotid gland	66	6 months	3 weeks	Mild
Present case	51	Female	Moderna (mRNA vaccine)	Once	Invasive ductal carcinoma	Breast	60	30 days	7 days	Mild

The actual incidence of RPP caused by the Moderna vaccine is unknown. It is impossible to conclude or predict the time required between RT and SARS-CoV-2 vaccination to prevent RRP. In addition, it is difficult to define the risk at the dose with RT of RPP caused by the Moderna mRNA vaccine. The limitation in the present study is that Fig. 3b, c are poor quality. The judgment from these figures that the improved skin condition has deteriorated after irradiation might be difficult. In addition, the effect of capecitabine could not be completely excluded.

This report is an interesting case report of RPP after administration of the mRNA-1273 SARS-CoV-2 vaccine (manufactured by Moderna). Because this study is a case study, it is difficult to define the indication of the mRNA-1273 SARS-CoV-2 vaccine for patients who have received RT.

## Data Availability

The data include individual patient data, but the data are available from the corresponding authors upon reasonable request.

## References

[1] D’angio GJ, Farber S, Maddock CL (1959). Potentiation of x-ray effects by actinomycin D. Radiology.

[2] Steber CR, Ponnatapura J, Hughes RT, Farris MK (2021). Rapid development of clinically symptomatic radiation recall pneumonitis immediately following COVID-19 vaccination. Cureus.

[3] Afacan E, Öğüt B, Üstün P (2021). Radiation recall dermatitis triggered by inactivated COVID-19 vaccine. Clin Exp Dermatol.

[4] Soyfer V, Gutfeld O, Shamai S (2021). COVID-19 vaccine-induced radiation recall phenomenon. Int J Radiat Oncol Biol Phys.

[5] Extermann M, Vogt N, Forni M, Dayer P (1995). Radiation recall in a patient with breast cancer treated for tuberculosis. Eur J Clin Pharmacol.

[6] Del Guidice SM, Gerstley JK (1988). Sunlight-induced radiation recall. Int J Dermatol.

[7] Azria D, Magné N, Zouhair A (2005). Radiation recall: a well recognized but neglected phenomenon. Cancer Treat Rev.

[8] Yeo W, Leung SF, Johnson PJ (1997). Radiation-recall dermatitis with docetaxel: establishment of a requisite radiation threshold. Eur J Cancer (Oxford, England: 1990).

[9] van Herpen CM, van Hoesel QG, Punt CJ (1995). Paclitaxel-induced severe hypersensitivity reaction occurring as a late toxicity. Ann Oncol.

[10] Stewart R, McDowell L (2021). Radiation recall phenomenon following COVID-19 vaccination. Int J Radiat Oncol Biol Phys.

